# Soluble AXL: A Possible Circulating Biomarker for Neurofibromatosis Type 1 Related Tumor Burden

**DOI:** 10.1371/journal.pone.0115916

**Published:** 2014-12-31

**Authors:** Gunnar Johansson, Po-Chun Peng, Po-Yuan Huang, Hsiung-Fei Chien, Kuo-Tai Hua, Min-Liang Kuo, Chin-Tin Chen, Ming-Jen Lee

**Affiliations:** 1 Department of Neurology, National Taiwan University Hospital, Taipei, Taiwan; 2 Institute for Systems Biology, Seattle, Washington, United States of America; 3 Department of Surgery, National Taiwan University Hospital, Taipei, Taiwan; 4 Graduate Institute of Toxicology, College of Medicine, National Taiwan University, Taipei, Taiwan; 5 Institute of Biochemical Science, College of Life Science, National Taiwan University, Taipei, Taiwan; 6 Department of Medical Genetics National Taiwan University Hospital, Taipei, Taiwan; University of Torino, Italy

## Abstract

Neurofibromatosis type 1 (NF1) is the most common tumor predisposition disorder affecting 1/3500 worldwide. Patients are at risk of developing benign (neurofibromas) and malignant peripheral nerve sheath tumors (MPNST). The AXL receptor tyrosine kinase has been implicated in several kinds of cancers, but so far no studies have investigated the role of AXL in NF1 related tumorigenesis. Recently, the soluble fraction from the extracellular domain of AXL (sAXL) has been found in human plasma, and its level was correlated to poor prognosis in patients with renal cancer. Compared to normal human Schwann cells, a significantly high expression level of AXL was found in three of the four MPNST cell lines and two of the three primary MPNST tissues. Similarly, the level of sAXL in conditioned media corresponded to the protein and mRNA levels of AXL in the MPNST cell lines. Furthermore, in two different human MPNST xenograft models, the human sAXL could be detected in the mouse plasma. Its level was proportionate to the size of the xenograft tumors, while no human sAXL was detect prior to the formation of the tumors. Treatment with a newly developed photodynamic therapy, prevented further tumor growth and resulted in drastically reduced the levels of sAXL compared to that of the control group. Finally, the level of sAXL was significantly increased in patients with plexiform tumors compared to patients with only dermal neurofibromas, further supporting the role of sAXL as a marker for NF1 related tumor burden.

## Introduction

Neurofibromatosis type 1 (NF1) is one of the most common autosomal dominant disorders affecting 1 in 3500 individuals world-wide. The clinical diagnostic criteria include café-au-lait spots, dermal or plexiform neurofibromas, Lisch nodules, optic glioma, bony defects and first degree relatives with NF1. In addition to skin pigmentation changes, the hallmark of NF1 is the formation of benign nerve sheath tumors along the peripheral nerves (neurofibromas). Neurofibromas are generally small localized tumors (dermal neurofibromas) that grow near the budding of the nerve. The dermal neurofibromas occur during childhood and grow progressively throughout life, especially during puberty and pregnancy [1]. While these tumors are found in virtually all patients with NF1, there are huge differences in the tumor burden even within family members with the same NF1 mutation. Patients can have anywhere between a few to several thousand tumors. The underlying reason to the differences remains unknown, but it suggests the involvement of epigenetic factors and/or modifier genes.

A more complex tumor is found in about one quarter of NF1 patients. The so called plexiform neurofibroma (pNFA) generally occurs before 2 years of age. In contrast to the dermal neurofibromas, pNFA often surrounds the entire nerve, and in some cases can grow extremely large covering entire limbs or large parts of the body. Total excision is often challenging due to their size and location. This is of particular concern as they can undergo malignant transformation and become a malignant peripheral nerve sheath tumor (MPNST) [2]. Patients with NF1 have a 5–13% life time risk of developing an MPNST, often occurring within a preexisting pNFA [3]. Given the difficulties for total excision of a large pNFA and no effective medication, markers that can detect and monitor the progression of pNFA is urgently needed.

The MPNST and neurofibroma cells differ from the normal Schwann cell in the expression of receptor tyrosine kinases (RTKs) [4], making them excellent candidates for drug interventions. The TAM (TYRO3, AXL and MER) family of receptors has been implicated in a wide range of functions including drug resistance, cell proliferation, cell adhesion and migration. TYRO3 is expressed predominantly within the nervous system, while AXL is ubiquitously expressed [5]. The TAM receptors are activated by a common ligand, GAS6 that has previously been shown to act as a mitogen for Schwann cells [6]. Recent studies showed that GAS6/AXL signaling directs neuronal migration via a signaling pathway involving RAS, RAC, p38MAPK, MAPKAP kinase 2, PI3K and HSP25 resulting in actin reorganization [7]. Up-regulation of AXL mRNA levels was demonstrated in one primary MPNST tumor [8], but the role of AXL in NF1 tumorigenesis is still unknown.

The kinase domain of AXL shares great similarities with the members of the MET tyrosine kinase family [9]. The MET and AXL receptors have been shown to activate similar downstream components [5]. Similar to MET, AXL is commonly up-regulated in drug resistant cell lines and cancers [10], [11]. In addition, MET is often upregulated in NF1 related tumors [4], [12], [13]. The extracellular portion of the AXL receptors consists of two immunoglobulin-like domains and two fibronectin type III domains, mimicking the structure of N-CAM [9]. The extracellular portion of AXL can be cleaved off from the membrane to generate soluble AXL (sAXL) [14], which is present in conditioned media from cancer cells as well as in human sera [14], [15]. In a recent publication, the levels of sAXL were reduced in patients with renal cancer compared to normal controls. In contrast high levels of sAXL correlated to worsen prognosis within the same patient group [16]. Herein, we set out to investigate the feasibility using soluble AXL as a marker to assess NF1 related tumor burden.

## Material and Methods

### Ethics statement

All MPNST cell lines used in this article have previously been published. For detailed information about ST8814, T265 and STS26T please refer to [17], for information about S462-TY and the establishment of the S462-TY xenograft model refer to [18]. The xenograft mouse models were in compliance with federal guidelines and under the approval of National Taiwan University Hospital's Animal Care and Use Committee. Human samples used in this study were obtained according to the principles expressed in the Declaration of Helsinki, approved by the Institutional Review Boards of the National Taiwan University Hospital and written informed consent was obtained from the patients.

### Primary cultures and MPNST cell lines

The S462-TY cell line was a kind gift from Dr. Timothy Cripe (Nationwide Childrens Hospital Columbus, OH) [18]. All other MPNST cell lines were a kind gift from Dr. Nancy Ratner (Cincinnati Children's Hospital, Cincinnati OH) and were grown as described [17], [19]. All MPNST cell lines, except STS26T was derived from NF1 patients. STS26T is a sporadic MPNST with no known NF1 mutations [20]. Primary neurofibroma cells were isolated immediately from resected tumor tissues as previously described [21]. The primary neurofibroma and the primary normal human Schwann cells (NHSC) (ScienCell Research laboratories, Carlsbad, CA) were grown on poly L lysine/laminin coated plates [17].

### Reagents

Rabbit anti AXL, mouse anti-beta-Actin and goat anti-TYRO3 antibodies were purchased from Santa Cruz Biotechnology (Santa Cruz Biotechnology, Inc., Santa Cruz, Ca). The Rabbit anti-phospho AXL was obtained from R&D Systems (R&D Systems, Inc., Minneapolis, Mn). We used three lentiviral shRNA against AXL: V2KHS_20187 (AXL-87), V2KHS_202535 (AXL-35) and V2KHS_238259 (AXL-59) (Open Biosystems Inc., Lafayette, Co) [22]. All shRNA cassettes were inserted in the GIPZ vector (Thermo Fisher Scientific Inc., Pittsburgh, PA), and we used the empty GIPZ vector as a negative control.

### Protein array analysis and Western blotting

The levels of 42 different phosphorylated RTKs were detected using a human phospho-RTK array kit from R&D Systems according to the manufacturer's protocol (R&D Systems, Inc., Minneapolis, MN). Protein extracts were prepared as previously described [23] from MPNST cell lines, ST8814, T265, STS26T and S462 as the Normal Human Schwann Cells (NHSC) grown in log phase in serum containing medium [17], [24]. For Western analysis, the protein samples were denatured in 6x SDS sample buffer (10% SDS, 30%glycerol, 0.6M DTT 0.012% bromophenol blue, 0.5 M Tris-HCl pH 6.8).

### Human plasma samples

The patients, fulfilled the diagnostic criteria of NF1, were recruited from the neurofibromatosis clinic in National Taiwan University Hospital, Taipei, Taiwan. Twenty milliliters of whole blood was drawn from the peripheral vein of participants. The samples in EDTA-coated tubes were centrifuged with initial spin at 2,200×*g* for 15 minutes followed by a further spin at 10,000×*g* for 5 minutes. The plasma was transferred and aliquoted before been stored in a −80°C freezer. Signed consent forms were obtained from all participants.

### Preparation of Lipo-Ce6

Chlorin E6 (Ce6), a natural product from live chlorella (Chlorella ellipsoidea), is an attractive photodynamic therapy (PDT) drug candidate since Ce6 has the high absorption in the red spectral region. It also exhibits advantageous photophysical properties for PDT such as having long lifetimes in their photoexcited triplet states and high molar absorption in the red region of the visible spectrum. As soon as Ce6 is exposed to specific wavelengths of light (≈662 nm), the Ce6-PDT generates reactive oxygen species leading to significant growth inhibition in malignant cells. In addition, Ce6-PDT induced apoptosis through the activation of caspase-3 and its downstream target, PARP cleavage [25]. Chlorine e6 (Ce6) was first encapsulated in liposomes by the film hydration. Briefly, in a 660 ml round bound flask, 0.2 mg of Ce6 in dimethylformamide (DMF) was added to 15.2 mmole of a lipid chloroform solution of DSPC (1,2-Distearoyl-sn-glycero-3-phosphocholine), cholesterol and DSPC-PEG (ratio of moles  = 50∶25∶1). After solvent removal by evaporation, a film was formed at the bottom of the flask to which 1 ml of 0.9% sodium chloride solution was added with further sonication for 20 minutes until the formation of liposome vesicles. The dispersion was then repeated 10 times passing through a polycarbonate membrane with a pore diameter of 100 nm in 65°C water bath. To purify the liposome-Ce6, the untrapped free Ce6 and lipids were removed by size exclusion chromatography using a Sephadex G-50 column.

### Characterization of liposomes

The amount of Ce6 in the liposome was measured by disrupting the liposome bilayer with absolute ethanol to release the entrapped Ce6. The concentration of Ce6 was determined by UV-visible spectroscopy at λ = 400 nm (DU800 Beckman Coulter, USA). The concentration of lipids in the liposomes was determined by the Bartlett assay [26]. Size distribution was measured with dynamic light scattering using a particle sizer (Coulter N4 Plus Submicron, Beckman Coulter, USA). In our preparations, Lipo-Ce6 contained about 18 µg of Ce6 per µmol of phospholipid. The particle size ranged from 115 to 125 nm in diameter.

### Xenograft mouse

Athymic nude mice (BioLASCO Co., Ltd,Taipei, Taiwan) were hosted in specific pathogen free environment with water and food *ad libitum*. Mice were subcutaneously injected with 1 million STS26T cells or 3 million S462-TY cells. Cells were dissolved in 1∶1 ratio of matrigel and Dulbecco's Modified Eagle's medium (DMEM) at a concentration of 5 or 15 million cells per ml. Mice were injected with 200 µl of the cell suspension into the left flank. At the endpoint of the study, animals were euthanized in accordance to the IACUC regulations. The experiment using STS26T was repeated twice, each time with 10 mice per group. To evaluate the correlation between the sAXL level and tumor size in non-treated mice, blood was withdrawn by cardiac puncture. A total of 20 tumor-bearing mice and 5 uninjected controls were used in the study.

For photodynamic treatment, the S462-TY tumors were allowed to grow up to 100 mm^3^ after which the mice were injected with 2 mg/kg or 2.5 mg/kg liposomes, Lipo-Ce6 dissolved in 0.9% normal saline. Control mice received a tail vein injection of 0.9% saline. Using a diode laser with power intensity of 105 mW/cm^2^ and 662 nm in wavelength, the light dose of 100 J/cm^2^ was illuminated upon the tumor two hours after the tail vein injection. Each treatment- and control- group consisted of four mice. The tumor size and body weight of the mice were measured every three days. The volume of tumor was calculated by the following formula: volume  = 6*Width^2^ × length/π. Fifty to one hundred microliters of blood was collected from the submandibular artery every six days. Blood was centrifuged and the plasma was stored in −80°C for ELISA measurement of sAXL.

### Detection of sAXL

The level of sAXL was measured using a commercial kit from R&D System (catalogue No. DY154). For each human and mouse sample, we used three independent concentrations in duplicates to ensure that the assay was measuring the concentration accurately. For the human study, the plasma was diluted with a range between 64 to 256 folds, while the mouse plasma was between 8 to 32 folds. After incubation overnight, we could detect the sAXL level at a concentration as low as 1 pg/ml with the dose dependent readings starting from 8 pg/ml (data not shown). To detect the sAXL in conditioned media, MPNST cell lines were grown in culture until 70% confluence. Plates were rinsed in PBS and serum free DMEM was added to the plates. Small samples of conditioned media were collected from each plate at the designated time points after adding the serum free media.

### Statistical Analysis

All statistical analysis were conducted by two-tailed Student's *t*-test with a *p-value* <0.05 as the statistical cutoff point.

## Results

### Expression of Receptor Tyrosine Kinases in MPNST cell lines

**Figure 1:**
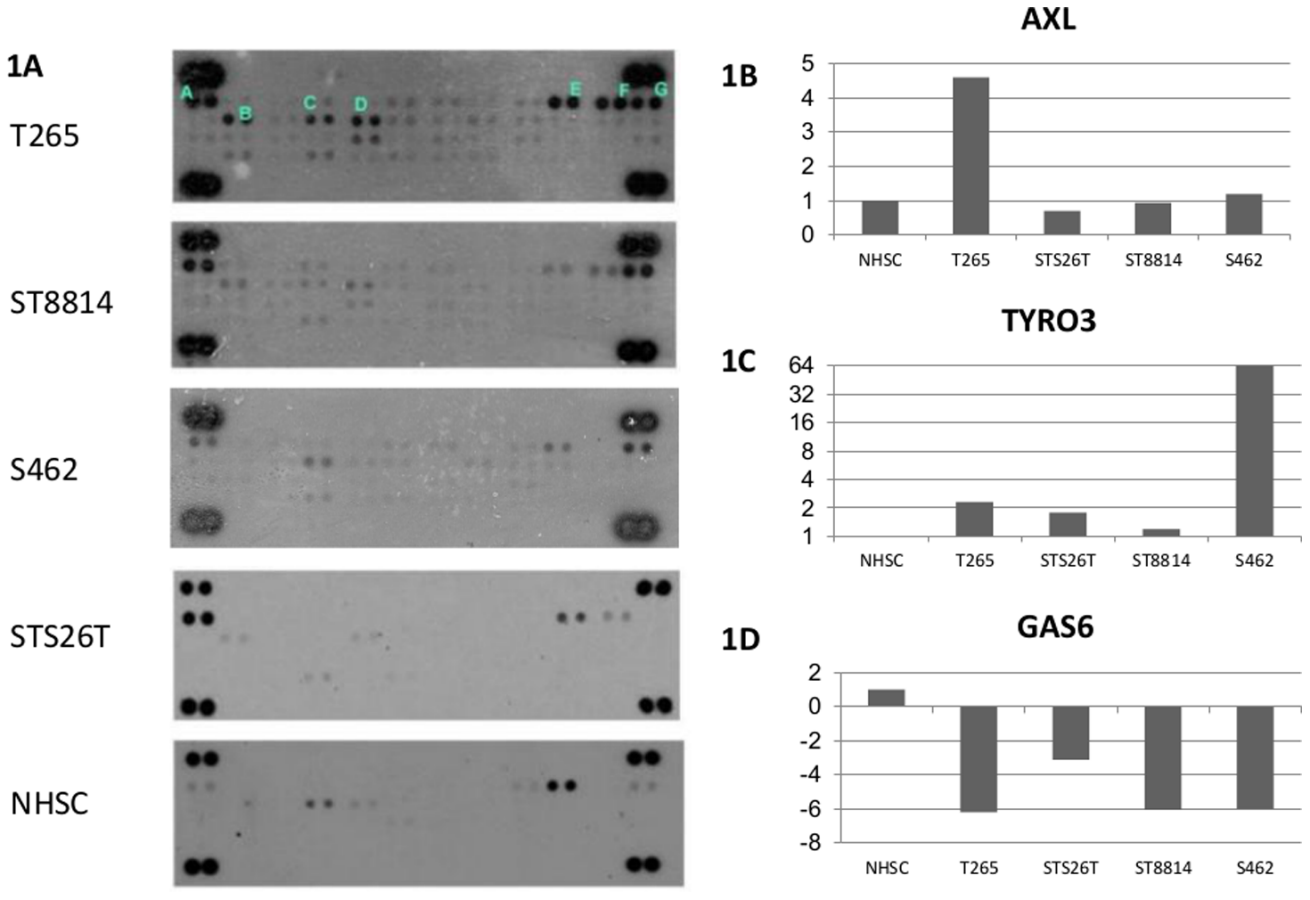
Increased phosphorylation activity of AXL and TYRO3 in MPNST cell lines. **A**, using a phospho-RTK array, the phosphorylation levels of 42 different RTKs in four MPNST cell lines (T265, STS26T, ST8814 and S462) and NHSC were measured. Seven of the RTKs had increased phosphorylation in MPNST cells: (**A**, EGFR; **B**, MET; **C**, PDGFR-alfa; **D**, PDGFR-beta; **E**, Insulin Growth factor receptor; **F**, AXL; **G**, TYRO3). **B∼1D**, mRNA expression of AXL (Fig. 1B), TYRO3 (Fig. 1C) and GAS 6 (Fig. 1D) compared to Normal Human Schwann cells (NHSC).

**Figure 2:**
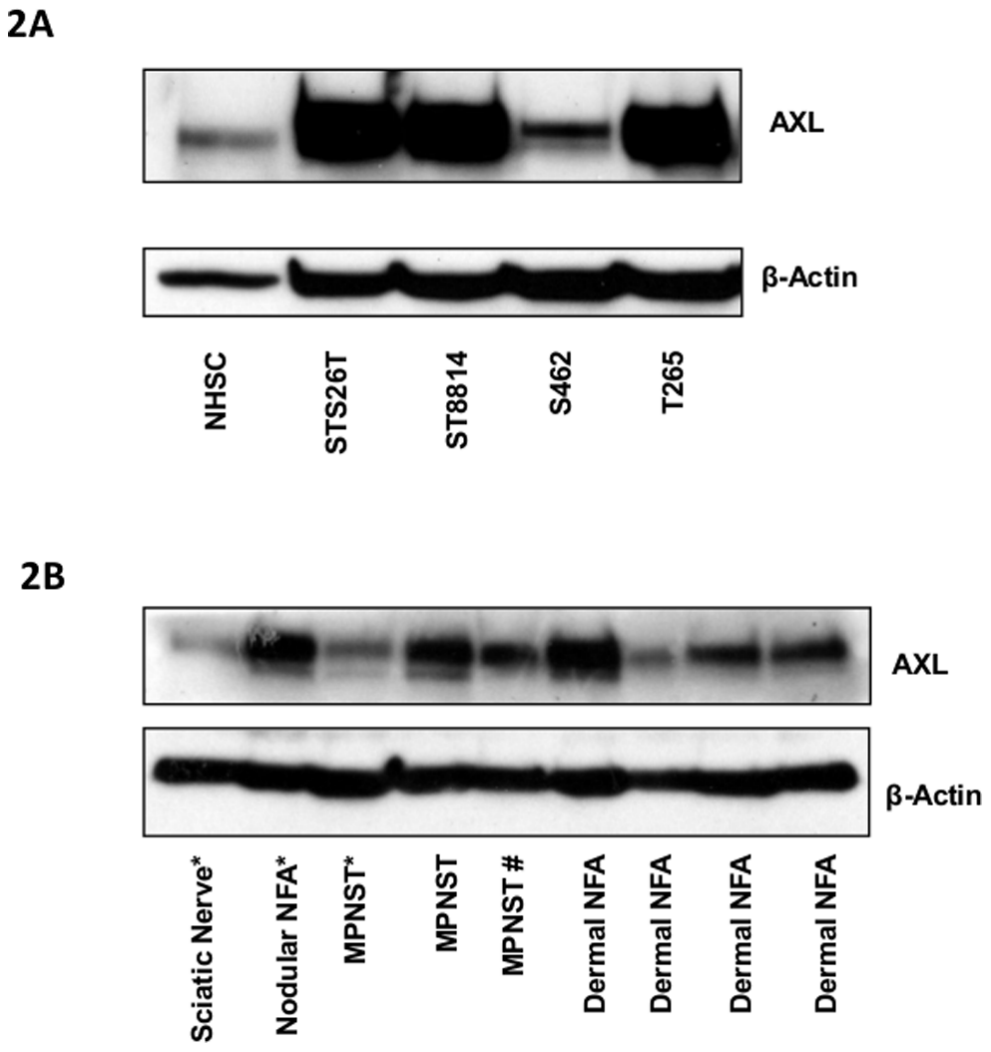
Increased protein expression of AXL in MPNST cell lines and human tumor samples. **A**, the expression of AXL protein in the four MPNST cells by Western blotting. **2B**, AXL protein expression in 3 MPNST samples, one nodular neurofibroma (a hard deeply located neurofibroma near the sciatic nerve), four dermal neurofibromas and the unaffected sciatic nerve from an NF1 patient was demonstrated by immunoblotting. Three of the samples marked with * were derived from the same patient. The other six samples are derived from six different patients. One MPNST sample marked with # was derived from a sporadic patient, without any family history or symptoms of NF1.

In order to evaluate the expression of receptor tyrosine kinases (RTKs) in MPNST cells, an array to detect phosphorylated Receptor Tyrosine Kinases was employed to screen the four MPNST cell lines and one batch of normal human Schwann cells (NHSC). Of the four cell lines used in our study, three were derived from familial cases of NF1 (ST8814, T265 and S462) and one was derived from a sporadic case of the disease (STS26T) [17]. The results from phosphorylated RTK protein array showed that the levels of phosphorylated EGFR, MET, PDGFRA, PDGFRB, AXL and TYRO3 were increased in at least three of the four MPNST cell lines tested (Fig. 1A). Two of these receptors, AXL and TYRO3 belong to the same family of receptors, and are activated by a common ligand GAS6. To further study the role of these receptors we evaluated their mRNA and protein expression using qPCR and Western analysis. In consistence with the results in Fig. 1A, three of the cell lines had increased protein levels of AXL compared to the NHSC (Fig. 2A). We only detected increased mRNA levels in one of these cell lines indicating that the increased phosphorylation seen in Fig. 1A could be due to post-transcriptional regulations (Fig. 1B). Interestingly, the cell line with the lowest level of AXL, S462, had the strongest upregulation of TYRO3 mRNA (Fig. 1C).However, expression of GAS6 mRNA encoding the ligand for AXL and TYRO3, was drastically decreased in all cell lines (Fig. 1D). In Western analysis, GAS6 protein was not detected in any of the MPNST cell lines (data not shown). Increased expression of AXL protein was also found in benign dermal neurofibromas (Fig. 2B). Likewise, strongly up-regulated AXL protein was also observed in the benign and malignant NF1 tumors compared to the unaffected peripheral nerve in the same NF1 patient (marked by a ‘*’ in Fig. 2B). Strong AXL expression was also observed in one MPNST and three out of four dermal neurofibroma samples that were derived from 5 different NF1 patients. In addition, we observed strong AXL expression in one MPNST specimen that was derived from a non-NF1 patient (marked #) (Fig. 2B).

### MPNST cell lines release soluble AXL into the conditioned media

**Figure 3:**
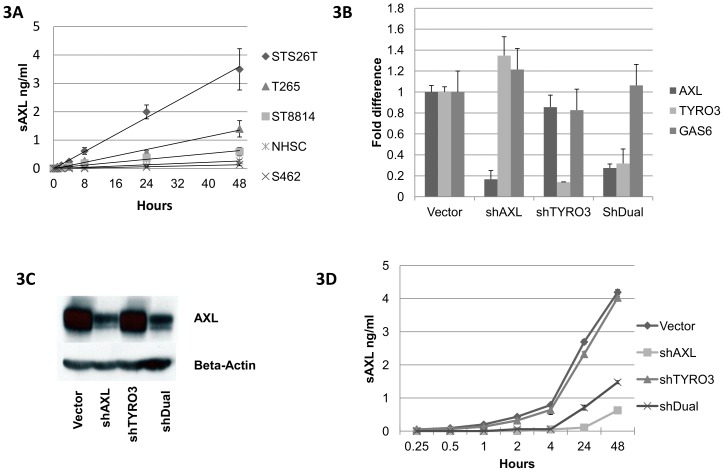
Release of sAXL into the conditioned media of MPNST cell lines. **A**, all four MPNST cell lines (S462, STS26T, ST8814 and T265) and NHSC released sAXL into the conditioned media, at levels that roughly comparable to the protein levels of full length AXL in the cells (Fig. 2A). A highly-matched correlation was found between the time in culture and the sAXL concentration in all four cell lines and NHSC (symbol cross for S462 cells with a coefficient of determination, R^2^ = 0.97; diamonds for STS26T with R^2^ = 0.99, squares for ST8814 with R^2^ = 0.97, triangles for T265 with R^2^ = 0.99 and star for NHSC R^2^ = 0.94). To further highlight that all cell lines release sAXL at a constant rate, the data in Fig. 3A is presented as 5 individual graphs in S1 Fig. **B–C**, Using specific lentiviral shRNA clones, the expression of AXL and TYRO3 has been knocked down in MPNST cell line T265, either alone or in combination to knockdown both genes (shDual). Successful silencing was verified with quantitative-PCR (Fig. 3B) and Western blotting (Fig. 3C). **D**, the levels of sAXL in the cell medium were drastically reduced in AXL-knockdown cells. In contrast, knockdown of TYRO3 did not affected the release of sAXL compared to the empty vector (GIPZ). In each case the level of sAXL in the conditioned media corresponded to the mRNA levels in the cells.

The AXL protein level is believed to be regulated in part by proteolytic cleavage of the extracellular portion to generate a soluble fraction of the protein [14], [15]. We grew our MPNST cell lines and NHSC to 70% confluence and replaced the media with serum free DMEM. At set time points, the conditioned media were collected to evaluate the level of soluble AXL using a human soluble AXL (sAXL) ELISA kit. The level of sAXL increased in a time-dependent manner and could be detected as early as 15 minutes after replacing the media. When assessing the level of sAXL in the medium by time, there was an almost perfect linear correlation indicating a constant rate of sAXL release from cells in culture (Fig. 3A and S1 Fig.). The S462 cell line and the NHSC had low but detectable levels of sAXL, which correlates well with the low levels of cellular AXL in these cells (Figs. 1B and 2A). As expected, knocking down AXL, or AXL in combination with TYRO3 (shDual) reduced the amount of sAXL in the conditioned media (Fig. 3D). The reduction of sAXL in the conditioned media corresponded to the reduction of AXL mRNA expression and the total protein levels in the tumor cell lines (Fig. 3B∼3D). In contrast, knockdown of TYRO3 did not affect the amount of sAXL in the conditioned media (Fig. 3B∼3D).

### Human sAXL can be detected in the plasma from xenograft mice

**Figure 4:**
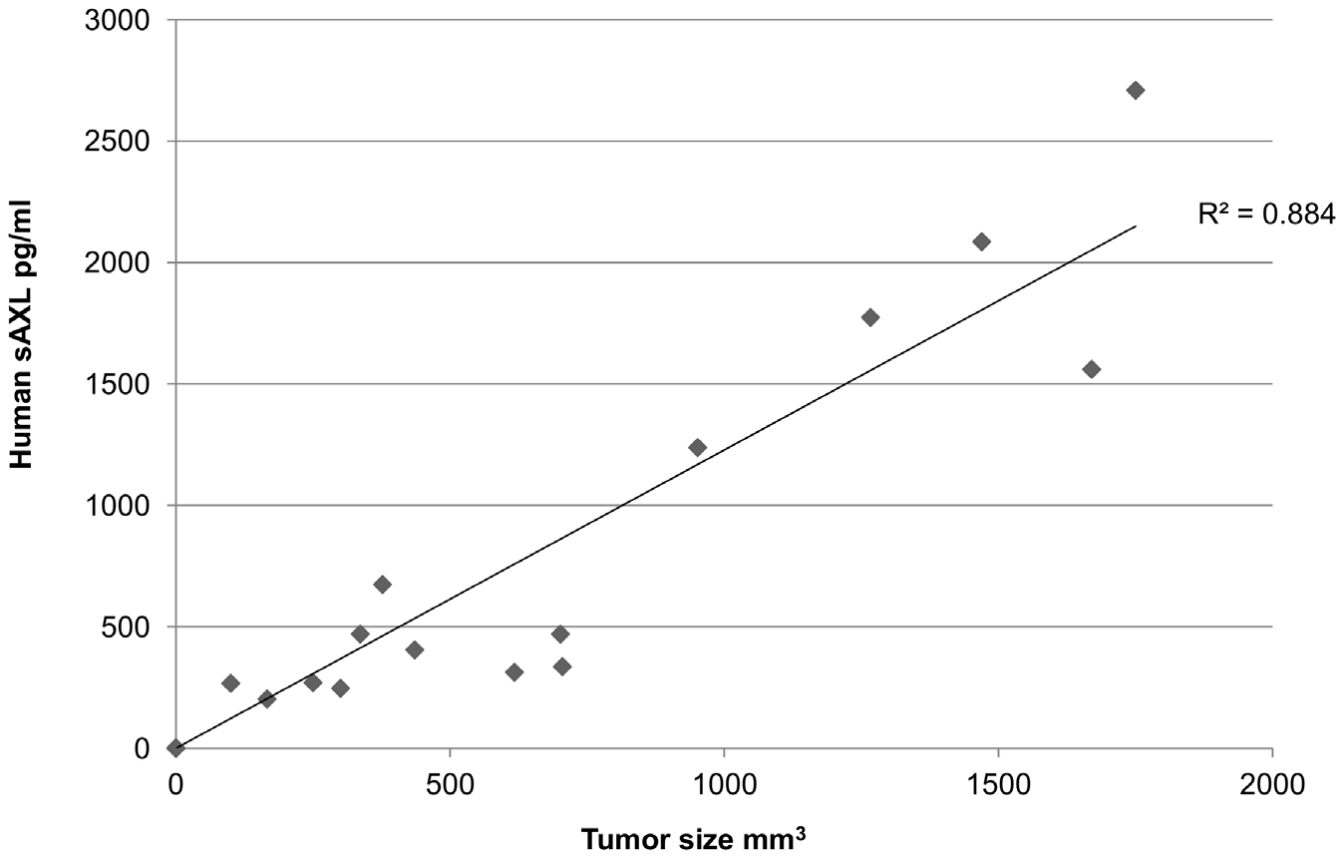
Mice harboring human MPNST (STS26T) xenograft tumors release human-soluble AXL into the blood. There was a trend of size dependent increasing of sAXL level until the tumors reached 2000 mm^3^. The coefficient of determination, R^2^ was 0.884.

To test the correlation between the sAXL and the MPNST tumor burden, we injected 19 mice with 1 million cells of the MPNST cell line, STS26T. Cells were dissolved in 1∶1 ratio of matrigel and DMEM at a concentration of 5 million cells per ml. Mice were injected with 200 µl of the cell suspension into the left flank. The plasma from the xenograft mice were collected by cardiac puncture at set time points. Human sAXL was detected in all the mice at a range of 200–2000 pg/ml (Fig. 4). In contrast, no sAXL was detected in any of the five non-engrafted control mice. Furthermore, the level of human sAXL correlated to the size of the tumor up to 2000 mm^3^ (the coefficient of determination, *R* ^2^ = 0.884) (Fig. 4).

**Figure 5:**
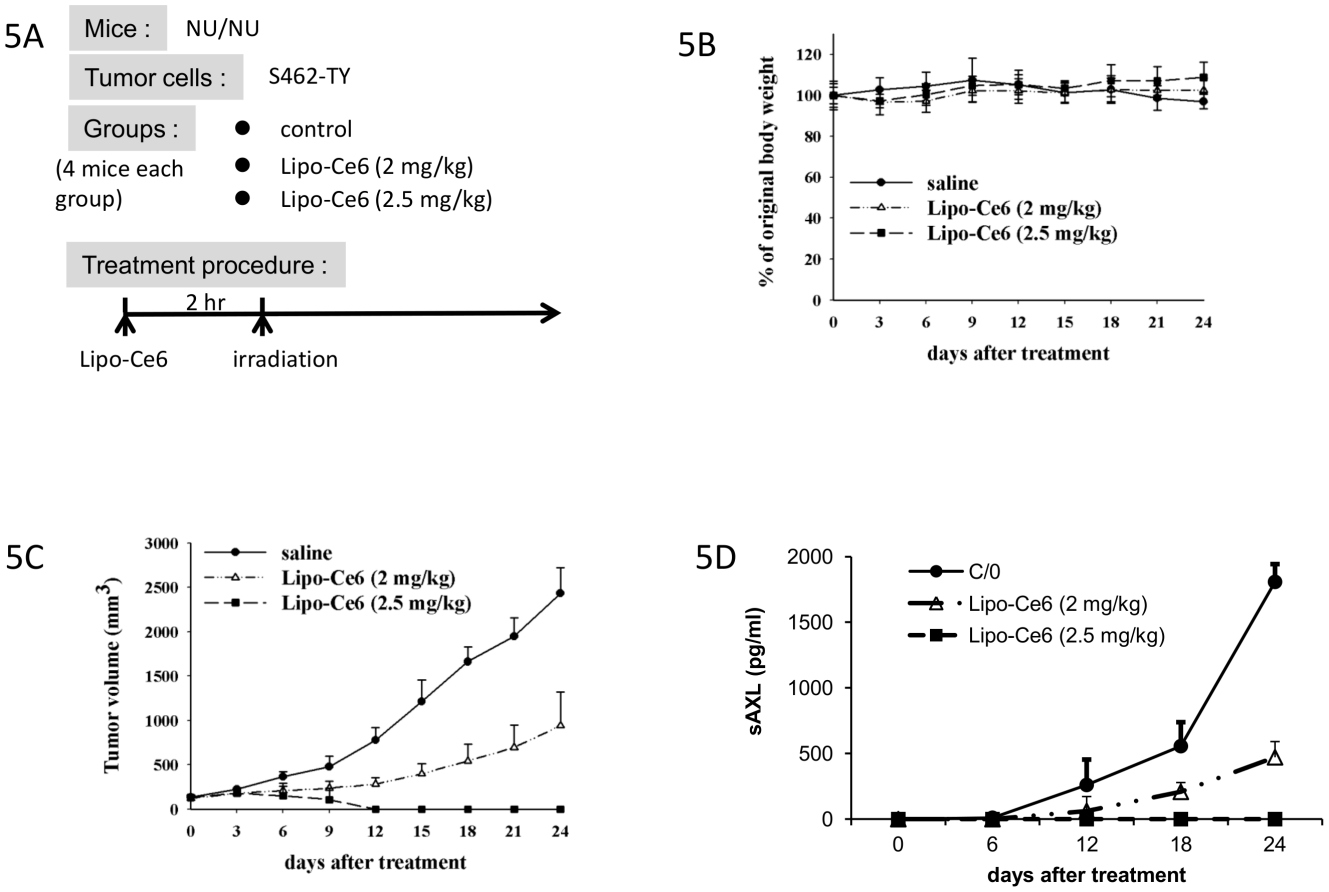
The plasma levels of human sAXL correlated to the tumor growth and the efficacy of Lipo-Ce6 in MPNST xenograft mice. **A**, MPNST xenograft tumors (S462-TY) were allowed to grow until 100 mm^3^. Mice were injected with normal saline, 2 mg/kg or 2.5 mg/kg Lipo-Ce6 by tail vein injection. Two hours post injection mice were irradiated with a light dose of 100 J/cm^2^. **B**, treatment had no adverse effect on the body weight. **C**, the tumor growth was completely suppressed in the mice receiving 2.5 mg/ml of Lipo-Ce6 followed by PDT treatment and partial suppression in the mice receiving 2 mg/kg Lipo-Ce6. **D**, the levels of sAXL corresponded to the tumor size in all three treatment groups further supporting its role as a marker for NF1 related tumor burden.

Lipo-Chlorine e6 (Ce6) photodynamic treatment (PDT) has previously been shown to be successful in treating gastric cancer cell lines [27]. Based on Lipo-Ce6 photodynamic treatment design [28], [29], the MPNST xenograft tumors were subjected to a photodynamic treatment (Manuscript in preparation) and their size was observed regularly. To verify the potential of using sAXL as a biomarker for tumor burden, blood from the treated mice was analyzed to assess the level of sAXL and to correlate with the tumor size (Fig. 5A). In each mouse, the level of sAXL increased by the size of the tumors (Fig. 5 C∼5D). Tumor progression was completely eliminated in mice receiving PDT with 2.5 mg/kg Lipo-Ce6 (Fig. 5C) and no sAXL was detected in their plasma (Fig. 5D). Meanwhile, mice receiving 2 mg/kg Lip-Ce6 PDT, the growth of tumors was retarded (Fig. 5C) and the levels of sAXL were reduced compared to the control group. These findings demonstrate a high correlation between the tumor burden and the plasma level of sAXL (Fig. 5D). Notably, treatment had no adverse effect on total body weight of the mice (Fig. 5B).

### Increased levels of soluble sAXL in patients with plexiform neurofibromas

**Figure 6:**
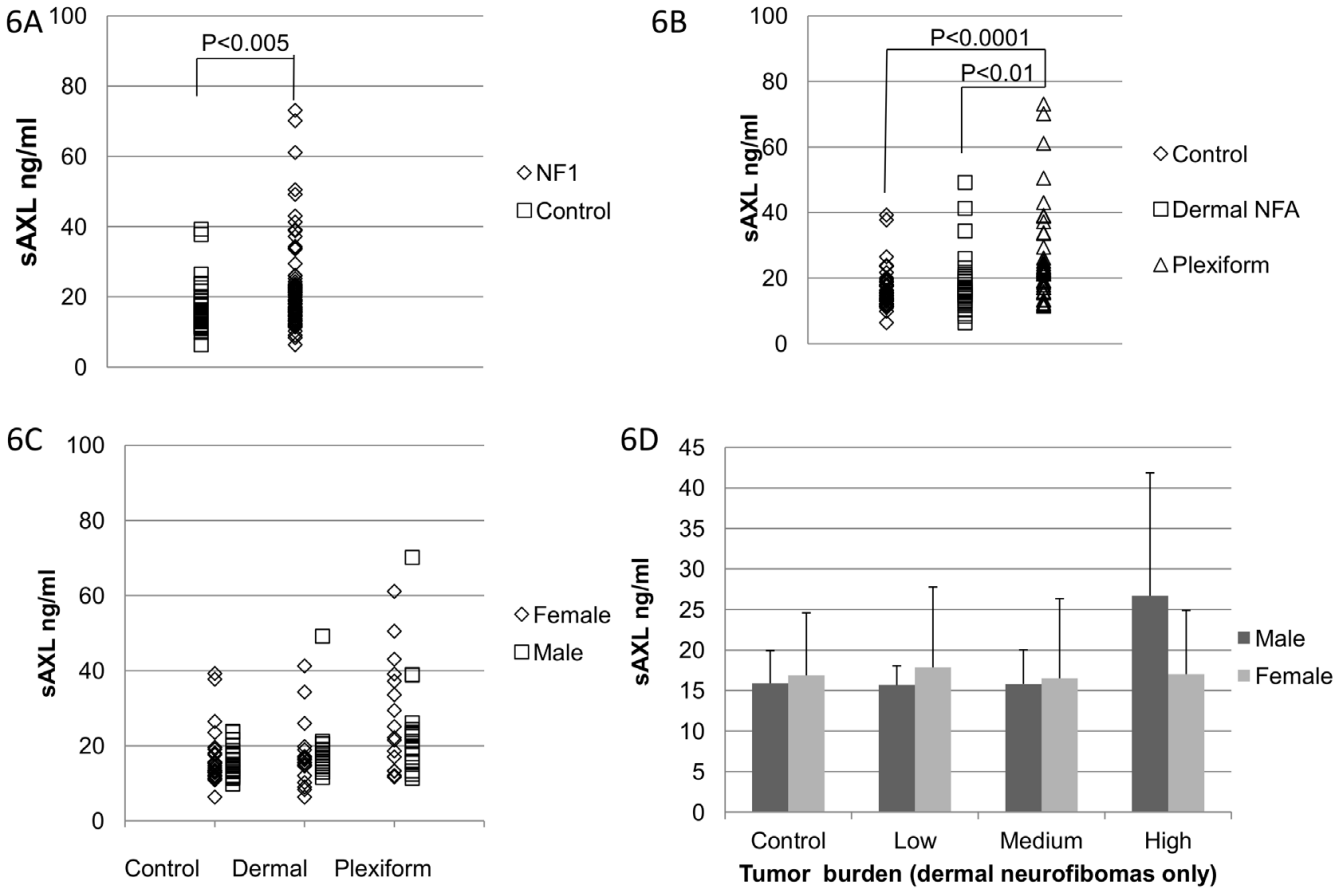
Increased plasma levels of sAXL in patients with plexiform neurofibromas. **A**, patients with neurofibromatosis had significantly higher plasma levels of sAXL. There were 72 NF1 patients (diamonds) and 46 controls (squares). The sAXL level was significantly higher in NF1 patients than that of controls (*p-value* <0.005). **B**, patients without plexiform tumors (group ‘Dermal NFA’, squares) had the same levels of sAXL compared to the healthy controls (group control, diamonds, n.s.  =  non-significant), while the levels were markedly increased in the patients with plexiform tumors (group ‘Plexiform’, triangles) compared to both the dermal NFA group (p<0.01) and the healthy control group (p<0.0001). **C**, there was not any significant difference between the male (squares) and female (diamonds) participants in any of the groups. **D**, within the dermal NFA group, males with a high number of dermal neurofibromas (>100) had significantly higher sAXL levels than that with a low NFA tumor burden (<30) (p<0.05). Nevertheless, no significant difference was found among female groups.

Given the correlation between tumor burden and plasma level of sAXL in mice, we further investigate whether these findings could be replicated in human. The levels of sAXL in 78 patients with NF1 and 46 healthy controls were evaluated by ELISA (S1–S3 Tables). The patients with malignancies were removed from the study, and the data from these patients are listed in a separate column in Table 1. The remaining 72 NF1 patients had significantly higher levels of sAXL (23±13 ng/ml) compared to the healthy control group (16±6 ng/ml) (*p*<0.01, Student's t-test, Fig. 6A). To differentiate the tumor burden in patients, we selected 36 NF1 patients with plexiform tumors, and 36 with only dermal neurofibromas for comparison. Interestingly, patients with plexiform neurofibromas had significantly higher levels of sAXL (26±16 ng/ml) compared to patients without plexiform neurofibromas (18±8 ng/ml) (*p*<0.01, Table 1 and Fig. 6B). In contrast, no statistically significant difference was seen between the non-plexiform group and healthy control (16±6 ng/ml) (Table 1 and Fig. 6B).

**Table 1:** Demographic data, tumor burden and level of soluble AXL in patients with neurofibromatosis type 1.

	NF1 patients without plexiform Neurofibroma		NF1 patients with plexiform Neurofibroma		NF1 patients with Cancer	
**Gender**	**Female**	**Male**	**Female**	**Male**	**Female**	**Male**
**Age (yrs)**	**34.1±10.4**	**33.1±13.4**	**40.5±14.6**	**32.6±11.0**	**46±10**	**35.4±5.8**
**Dermal NFAs**						
** <30**	**8**	**10**	**2**	**3**	**1**	**2**
** 30∼100**	**3**	**3**	**4**	**7**	**0**	**3**
** >100**	**8**	**4**	**10**	**10**	**1**	**0**
**sAXL conc. (ng/ml)**	**14.2±3.8**	**18.2±8.5**	**28.1±14.4**	**23.7±13.2**	**45.1±28.0**	**23.7±5.5**

Dermal NFAs  =  number of skin neurofibromas; sAXL conc.  =  the concentration of soluble AXL; yrs  =  years.

To exclude other confounding factors, the effect of gender, age and the number of dermal neurofibromas were also analyzed (S1–S2 Tables). Interestingly, the female patients had a more even distribution in all three groups, while the male patients were clustered at comparably lower concentrations with a few outliers with high levels of sAXL (Fig. 6C). To further evaluate this phenomenon we divided the non-plexiform group into three subgroups based on an estimation of their tumor burden. Estimation was done based on consulting the relevant physician. Patients with less than 30 visible tumors were defined as low tumor burden (n = 8 males/10 females). Patients with several hundred of tumors were defined as high burden (n = 4 males/8 females), and patients in between received a medium score (n = 3 males/3 females). In male patients' data, high tumor burden correlated with an increased sAXL concentration patients (p<0.05), while no such correlation was seen in the female patients (p<0.05) (Table 1, Fig. 6D).

## Discussion

The MPNST and neurofibroma cells differ from the normal Schwann cells in the expression of receptor tyrosine kinases (RTKs) [4], making them excellent candidates for drug interventions. So far most of the studies have focused on a few selected RTKs such as EGFR and PDGFR, but the clinical trials using drugs that target these receptors have had limited effects [30], [31]. Here we utilized a phospho-RTK array measuring the activity of 42 different RTKs. In addition to EGFR [32] and PDGFR [33] that has previously been reported in the context of NF1, we found increased phosphorylation of AXL in the MPNST cell lines.

The AXL receptor tyrosine kinase was up-regulated in benign and malignant nerve sheath tumors compared to normal Schwann cells (Figs. 1 and 2). The soluble form of AXL (sAXL) was found in conditioned media of MPNST cells (Fig. 3) and in the plasma from mice harboring human MPNST xenograft tumors (Figs. 4 and 5). Moreover, the level of human sAXL in mice correlated to the size of the tumors, while there was no human sAXL detected in uninjected mice (Fig. 4). We verified these data in a second xenograft model, in which mice were treated with photodynamic therapy (PDT) using an increasing concentration of Lipo-Chlorine Ce6. As expected, the level of sAXL correlated to the tumor size in all three treatment groups, further emphasizing the role of sAXL as a biomarker for tumor burden. Finally, the level of sAXL was elevated in NF1 patients with plexiform tumors compared to patients with only dermal neurofibromas and controls. With a trend towards a high level of sAXL in patients with high number of dermal neurofibromas compared to that of the patients with fewer tumors (Fig. 6).

The expression difference of AXL between benign and malignant tumors is still unclear. One of the patients in the study had his leg amputated in order to remove the Plexiform/MPNST tumor. He donated the leg to our study and we obtained Plexiform/MPNST tumor, benign neurofibromas and normal sciatic nerve tissues from his leg. In this patient we found strong expression of AXL in the excised nodular neurofibroma, while the plexiform/MPNST tumor from the same patient had lower levels; however, it was still higher than that of the normal sciatic nerve. Histological examination of the Plexiform/MPNST tumor, showed a malignant transformation in some parts, and it is unclear whether the part that was analyzed in this experiment had malignant or benign cells. The other two MPNST samples (from two other patients) in this study had high levels of AXL, as did 1 out 4 dermal neurofibromas (Fig. 2B). The other three dermal neurofibromas had intermediate levels between the sciatic nerve and the malignant tumors (Fig. 2B).

Several studies have shown that soluble cell surface receptors like platelet-derived growth factor receptor and epidermal growth factor receptor can be detected in both conditioned media from cancer cells and patient plasma; however, it has not been clearly demonstrated whether these soluble cell surface receptors in the plasma are actually originated from the tumor cells [16], [34], [35]. In one study sAXL was detected in the tumor exudates of xenograft mice [36]; however, the authors used an antibody that detected both murine and human AXL. Hence, the origin of sAXL was not addressed. Furthermore, it has been argued that the increased levels of sAXL seen in the patients did not originate from the tumor cells. The claim was solely based on the lack of correlation between the tumor size and serum levels of AXL in renal cancer patients [16].

In our study the levels of sAXL seem to be correlated with the level of AXL in MPNST cells (Figs. 1–3). In all four MPNST cell lines as well as in the NHSC, the release of sAXL was maintained at constant rate over time in serum free media. As expected, the release of sAXL was the lowest in S462 and NHSC corresponding to the relatively low levels of cellular AXL. (Figs. 2A, 3A and S1 Fig.). Further, knockdown of AXL reduced the levels of sAXL to the same extend as the reduction of AXL mRNA and protein levels (Fig. 3B–3D). Furthermore, human sAXL can be detected in the plasma from xenograft mice. Treatment with photodynamic Lipo-ce6 reduced the tumor size and the sAXL levels accordingly. Taken together these findings argue that the sAXL in the plasma originates from the tumor cells, and it might be useful to evaluate sAXL as tumor burden marker in NF1 patients.

The role of AXL in the MPNST cells is still unclear. In our study, silencing the expression of AXL did not affect cell proliferation or the subcutaneous tumor growth, but resulted in a slight but significant down regulation of cell migration (data not shown). In epithelial ovarian cancer, AXL was over-expressed in the advanced metastatic tumors compared to the low grade tumors. The authors noticed reduced tumor growth after intraperitoneal injections of the tumor cells and an adenovirus carrying sAXL was able to reduce the growth of these xenograft tumors [37]. In addition, GAS6-AXL signaling has recently been shown to increase Schwannoma cell matrix adhesion and survival [38], further arguing for an involvement of AXL in Schwann cell tumorigenesis.

Interestingly, the levels of sAXL seem to be more evenly distributed in the female patients compared to the male patients (Fig. 6C–6D, S1 and S2 Tables). In the male dermal neurofibroma patients, one patient stood out with 49 ng/ml sAXL compared to the other 16 patients that had between 11.5–20.6 ng/ml. This patient had extreme numbers of neurofibromas, including spinal tumors along all major nerves (Fig. 6C, S1 Table). Hence, a high sAXL level in this patient supports the general idea of sAXL as tumor burden marker. Within the dermal neurofibroma group, the four male patients with more than 100 dermal neurofibromas, had significant higher sAXL levels than the ten male patients with less than 30 dermal neurofibromas (p<0.05). As a group these high dermal tumor burden patients had comparable levels to the male plexiform patients (Fig. 6C–6D).

In contrast, some of the mild female NF1 patients and even some of the female controls had high levels of sAXL (Fig. 6C, S1 Table). This may be caused by the menstrual cycle and hormonal changes on sAXL levels. In previous report, GAS6-AXL signaling has been proposed to regulate the migration of Gondotropin-Releasing Hormone (GnRH) neurons, an essential process during sexual maturation [7]. However, the potential effect of sAXL on hormonal release in adults is not well understood.

Plasma samples from five patients with MPNST and one with glioblstoma multiforme was analyzed (Table 1, S3 Table). As a group, the levels of sAXL did not deviate from the levels of the plexiform tumors. The patient with glioblastoma had the highest sAXL level in the study (73.1 ng/ml), while the five MPNST patients had levels that were similar to the plexiform patients (ranging from 17.1–33.9 ng/ml) (Table 3). One of the low scoring MPNST patients had the plasma drawn after surgically removing all visible sign of the tumor. The other four patients had parts of the tumors removed and were undergoing chemotherapy at the time of the plasma collection. It is unclear to what extent the ongoing therapy affected the release of AXL. Comparing the sAXL levels before and after the excision of a large plexiform neurofibroma or an MPNST would be informative on the role of sAXL as marker for these tumors.

In conclusion, we report an increased expression and phosphorylation of AXL in MPNST cells with a corresponding increase in levels of sAXL in the medium when the tumor cells were grown in culture and in the mouse plasma when injected into nude mice. Further, *in vivo* and human studies confirmed the high correlation between the tumor burden in NF1 disease and the plasma level of sAXL. Therefore, monitoring the plasma level of sAXL may provide a useful reference for tumor growth and an accurate monitoring for treatment efficacy in NF1 patients with a plexiform neurofibroma or MPNST.

## Supporting Information

S1 Fig **Release of sAXL in MPNST cell lines (STS26T, ST8814, S462, and T265) and normal human Schwann cells (NHSC).** Data from Fig. 3A has been separated into 5 different graphs to highllight that sAXL is released at a constant rate in all cell lines and NHSC. The Y-Axis is the level of sAXL (ng/ml) release by the cells and the X-Axis represents how long the duration (hours) of the cells in serum free media.
(PPTX)

S1 Table **NF1 patients without plexiform Neurofibroma.** Age  =  age in years; Avg.  =  average of the levels of plasma soluble AXL; F =  female; M =  male; Patient nr.  =  patient number; pNFA  =  growth of plexiform neurofibroma; sAXL  =  plasma levels of soluble AXL; SD  =  standard deviation of the levels of plasma soluble AXL; #NFA  =  number of skin neurofibroma.
(DOCX)

S2 Table **NF1 patients with plexiform Neurofibroma.** Age  =  age in years; Avg.  =  average of the levels of plasma soluble AXL; F =  female; M =  male; Patient nr.  =  patient number; pNFA  =  growth of plexiform neurofibroma; sAXL  =  plasma levels of soluble AXL; SD  =  standard deviation of the levels of plasma soluble AXL; #NFA  =  number of skin neurofibroma.
(DOCX)

S3 Table **NF1 patients with cancer.** Age  =  age in years; Avg.  =  average of the levels of plasma soluble AXL; F =  female; M =  male; MPNST  =  growth of malignant peripheral nerve sheath tumor; Patient nr.  =  patient number; pNFA  =  growth of plexiform neurofibroma; sAXL  =  plasma levels of soluble AXL; SD  =  standard deviation of the levels of plasma soluble AXL; #NFA  =  number of skin neurofibroma.
(DOCX)
