# Correction: Soluble AXL: A Possible Circulating Biomarker for Neurofibromatosis Type 1 Related Tumor Burden

**DOI:** 10.1371/journal.pone.0119975

**Published:** 2015-03-17

**Authors:** The PLOS ONE Staff

The affiliation for the second and seventh authors is incorrect. Po-Chun Peng and Chin-Tin Chen are not affiliated with #2 but with #5 Institute of Biochemical Science, College of Life Science, National Taiwan University, Taipei, Taiwan.
