# Supplementary material for: Soluble AXL: A Possible Circulating Biomarker for Neurofibromatosis Type 1 Related Tumor Burden
Source: PLoS One. 2014 Dec 31;9(12):e115916. doi: 10.1371/journal.pone.0115916 (PMC4281253; doi:10.1371/journal.pone.0115916)
Supplement: S1 Fig — Release of sAXL in MPNST cell lines (STS26T, ST8814, S462, and T265) and normal human Schwann cells (NHSC). Data from Fig. 3A has been separated into 5 different graphs to highllight that sAXL is released at a constant rate in all cell lines and NHSC. The Y-Axis is the level of sAXL (ng/ml) release by the cells and the X-Axis represents how long the duration (hours) of the cells in serum free media. (PPTX) [file pone.0115916.s001.pptx]

## Slide 1
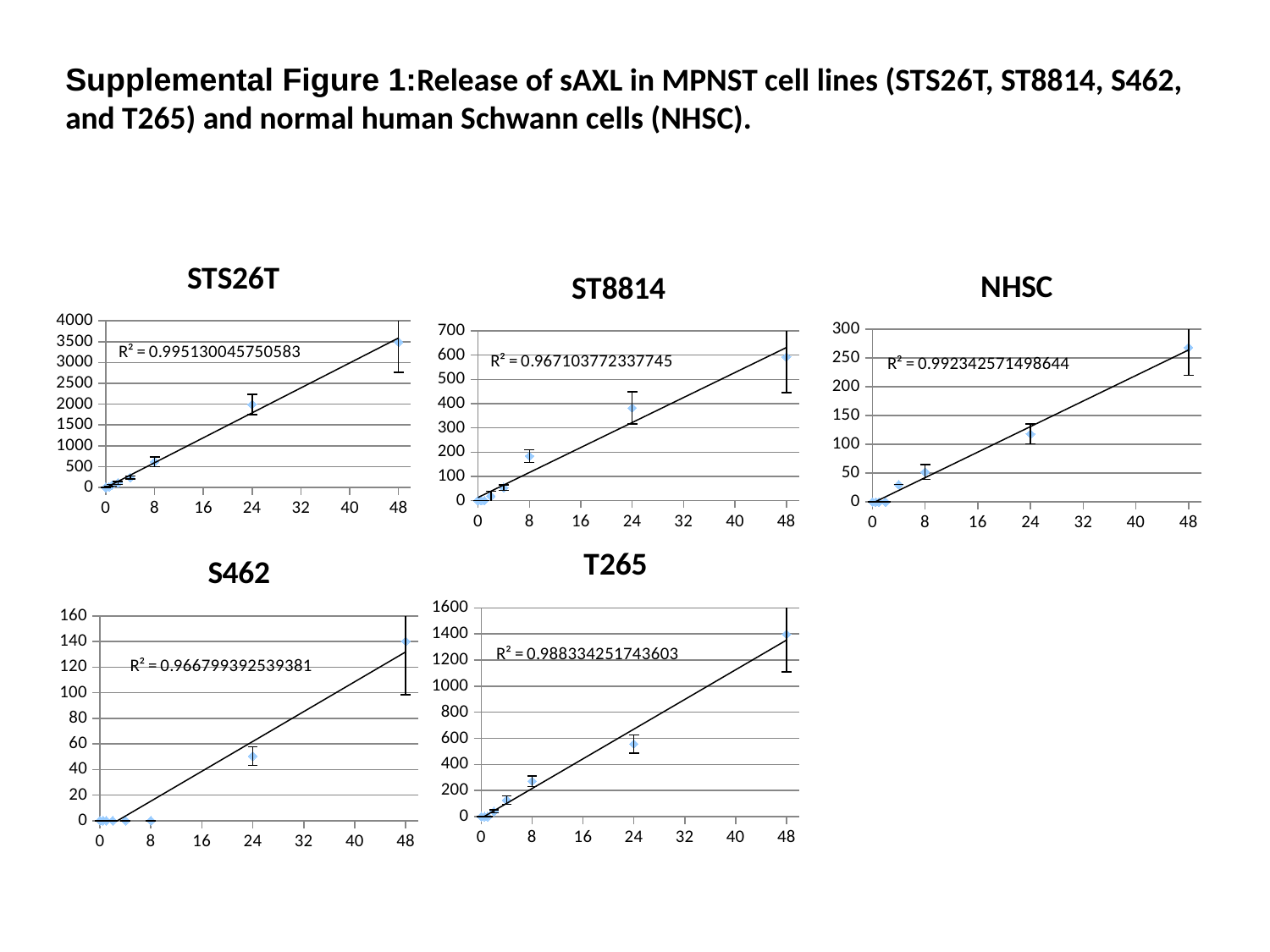

Supplemental Figure 1:Release of sAXL in MPNST cell lines (STS26T, ST8814, S462, and T265) and normal human Schwann cells (NHSC).
### Chart:
| Category | STS26T |
|---|---|
### Chart: NHSC
| Category | |
|---|---|
### Chart:
| Category | ST8814 |
|---|---|
### Chart:
| Category | T265 |
|---|---|
### Chart:
| Category | S462 |
|---|---|
