# Supplementary material for: Soluble AXL: A Possible Circulating Biomarker for Neurofibromatosis Type 1 Related Tumor Burden
Source: PLoS One. 2014 Dec 31;9(12):e115916. doi: 10.1371/journal.pone.0115916 (PMC4281253; doi:10.1371/journal.pone.0115916)
Supplement: S1 Table — NF1 patients without plexiform Neurofibroma. Age = age in years; Avg. = average of the levels of plasma soluble AXL; F = female; M = male; Patient nr. = patient number; pNFA = growth of plexiform neurofibroma; sAXL = plasma levels of soluble AXL; SD = standard deviation of the levels of plasma soluble AXL; #NFA = number of skin neurofibroma. (DOCX) [file pone.0115916.s002.docx]

**Supplemental Tables**

**Table S1: NF1 patients without plexiform Neurofibroma**

| **Patient nr** | **Age** | **Sex** | **# NFA** | **pNFA** | **sAXL (ng/ml)** |  | **Patient nr** | **Age** | **Sex** | **# NFA** | **pNFA** | **sAXL (ng/ml)** |
| --- | --- | --- | --- | --- | --- | --- | --- | --- | --- | --- | --- | --- |
| NF260 | 36 | F | <30 | No | 41.3 |  | NF247 | 26 | M | >100 | No | 49.2 |
| NF290 | 29 | F | >100 | No | 34.3 |  | NF 43 | 63 | M | >100 | No | 21.2 |
| NF114 | 34 | F | >30 | No | 26.0 |  | NF125 | 31 | M | >30 | No | 20.7 |
| NF223 | 35 | F | >100 | No | 19.8 |  | NF 104 | 55 | M | >100 | No | 20.4 |
| NF 054 | 43 | F | >100 | No | 14.6 |  | NF355 | 14 | M | <10 | No | 19.1 |
| NF 248 | 23 | F | <30 | No | 12.1 |  | NF 271-1 | 30 | M | <30 | No | 18.1 |
| NF 251 | 26 | F | <30 | No | 9.1 |  | NF162 | 28 | M | <10 | No | 18.0 |
| NF 108 | 47 | F | >100 | No | 8.4 |  | NF288 | 46 | M | <30 | No | 16.5 |
| NF 256 | 28 | F | <30 | No | 19.0 |  | NF422 | 33 | M | >100 | Removed | 16.0 |
| NF385 | 42 | F | >30 | No | 6.4 |  | NF 190 | 21 | M | <30 | No | 15.8 |
| NF389 | 30 | F | <30 | No | 15.5 |  | NF 284 | 35 | M | <30 | No | 14.6 |
| NF15 | 20 | F | <30 | No | 14.8 |  | NF 266 | 26 | M | <10 | No | 14.4 |
| NF134 | 54 | F | >100 | No | 16.8 |  | NF90-1 | 11 | M | <10 | No | 13.6 |
| NF301 | 21 | F | <30 | No | 15.0 |  | NF 138 | 52 | M | >30 | No | 13.8 |
| NF369 | 24 | F | <30 | No | 16.3 |  | NF 237 | 30 | M | <30 | No | 13.1 |
| NF372 | 37 | F | >100 | No | 15.5 |  | NF373 | 29 | M | >30 | No | 13.0 |
| NF 268 | 39 | F | >100 | No | 16.3 |  | NF376 | 33 | M | <30 | No | 11.6 |
| NF379 | 24 | F | >30 | No | 17.2 |  |  |  |  |  |  |  |
| NF378 | 56 | F | >100 | No | 10.3 |  |  |  |  |  | **Avg** | **18.2** |
|  |  |  |  | **Avg** | **14.2** |  |  |  |  |  | **SD** | **8.5** |
|  |  |  |  | **SD** | **3.8** |  |  |  |  |  |  |  |
