# Supplementary material for: Soluble AXL: A Possible Circulating Biomarker for Neurofibromatosis Type 1 Related Tumor Burden
Source: PLoS One. 2014 Dec 31;9(12):e115916. doi: 10.1371/journal.pone.0115916 (PMC4281253; doi:10.1371/journal.pone.0115916)
Supplement: S2 Table — NF1 patients with plexiform Neurofibroma. Age = age in years; Avg. = average of the levels of plasma soluble AXL; F = female; M = male; Patient nr. = patient number; pNFA = growth of plexiform neurofibroma; sAXL = plasma levels of soluble AXL; SD = standard deviation of the levels of plasma soluble AXL; #NFA = number of skin neurofibroma. (DOCX) [file pone.0115916.s003.docx]

**Supplemental Tables**

**Table S2: NF1 patients with plexiform Neurofibroma**

| **Patient nr** | **Age** | **Sex** | **# NFA** | **pNFA** | **sAXL (ng/ml)** |  | **Patient nr** | **Age** | **Sex** | **# NFA** | **pNFA** | **sAXL (ng/ml)** |
| --- | --- | --- | --- | --- | --- | --- | --- | --- | --- | --- | --- | --- |
| NF323 | 25 | F | <30 | Yes | 61.1 |  | NF262 | 29 | M | <30 | Yes | 70.1 |
| NF325-1 | 64 | F | >100 | Yes | 50.5 |  | NF 153 | 52 | M | >100 | Yes | 39.0 |
| NF305 | 24 | F | <100 | Yes | 43.0 |  | NF294 | 13 | M | >30 | Yes | 38.8 |
| NF331 | 56 | F | >100 | Yes | 37.2 |  | NF309 | 32 | M | >100 | Yes | 26.1 |
| NF 7 | 36 | F | <30 | Yes | 29.5 |  | NF 059 | 50 | M | >100 | Yes | 23.6 |
| NF334 | 69 | F | >100 | Yes | 25.2 |  | NF325 | 38 | M | >100 | Yes | 23.3 |
| NF 196 | 36 | F | >100 | Yes | 33.6 |  | NF 276 | 12 | M | <30 | Yes | 22.7 |
| NF 224 | 25 | F | >100 | Yes | 12.1 |  | NF295 | 18 | M | >100 | Yes | 21.2 |
| NF271 | 57 | F | >100 | Yes | 18.6 |  | NF 146 | 35 | M | >30 | Yes | 21.2 |
| NF 332 | 36 | F | >100 | Yes | 39.1 |  | NF 029 | 28 | M | <30 | Yes | 17.9 |
| NF 398 | 30 | F | >30 | Yes | 22.2 |  | NF 200 | 38 | M | >30 | Yes | 15.9 |
| NF313 | 17 | F | >30 | Yes | 21.6 |  | NF 109 | 41 | M | >100 | Yes | 15.5 |
| NF314 | 44 | F | >100 | Yes | 13.3 |  | NF 246 | 28 | M | >100 | Yes | 13.3 |
| NF193 | 41 | F | >100 | Yes | 17.1 |  | NF 253 | 29 | M | >50 | Yes | 11.4 |
| NF22 | 40 | F | >100 | Yes | 11.7 |  | NF 72-1 | 23 | M | >50 | Yes | 19.2 |
| NF287 | 48 | F | >30 | Yes | 13.3 |  | NF308 | 40 | M | >100 | Yes | 19.1 |
|  |  |  |  | **Avg** | **28.1** |  | NF352 | 26 | M | >50 | Yes | 15.7 |
|  |  |  |  | **SD** | **14.4** |  | NF367 | 51 | M | >100 | Yes | 24.3 |
|  |  |  |  |  |  |  | NF249 | 33 | M | >50 | Yes | 12.4 |
|  |  |  |  |  |  |  | NF309 | 35 | M | >100 | Yes | 23.1 |
|  |  |  |  |  |  |  |  |  |  |  | **Avg** | **23.7** |
|  |  |  |  |  |  |  |  |  |  |  | **SD** | **13.2** |
