# Supplementary material for: Soluble AXL: A Possible Circulating Biomarker for Neurofibromatosis Type 1 Related Tumor Burden
Source: PLoS One. 2014 Dec 31;9(12):e115916. doi: 10.1371/journal.pone.0115916 (PMC4281253; doi:10.1371/journal.pone.0115916)
Supplement: S3 Table — NF1 patients with cancer. Age = age in years; Avg. = average of the levels of plasma soluble AXL; F = female; M = male; MPNST = growth of malignant peripheral nerve sheath tumor; Patient nr. = patient number; pNFA = growth of plexiform neurofibroma; sAXL = plasma levels of soluble AXL; SD = standard deviation of the levels of plasma soluble AXL; #NFA = number of skin neurofibroma. (DOCX) [file pone.0115916.s004.docx]

**Supplemental Tables**

**Table S3: NF1 patients with cancer**

| **Patient nr** | **Age** | **Sex** | **#NFA** | **pNFA** | **Cancer** | **sAXL (ng/ml)** |  |
| --- | --- | --- | --- | --- | --- | --- | --- |
| NF39-2 | 36 | F | <30 | Yes | Brain | 73.1 | Malignant glioma |
| NF272 | 27 | M | <30 | Yes | MPNST | 33.9 |  |
| NF316 | 38 | M | >30 | Yes | MPNST | 23.0 | All identified MPNST removed |
| NF261 | 41 | M | >30 | Yes | MPNST | 22.5 |  |
| NF 399 | 30 | M | >30 | Yes | MPNST | 21.9 |  |
| NF267 | 56 | F | >100 | Yes | MPNST | 17.1 |  |
| NF316 | 41 | M | <30 | Yes | MPNST | 17.1 |  |
|  |  |  |  |  | **Avg** | **29.8** |  |
|  |  |  |  |  | **SD** | **19.9** |  |
